# Mechanisms of Action of Herbal Preparations for the Rehabilitation of Dust-Related Diseases of the Bronchopulmonary System

**DOI:** 10.3390/biomedicines14091936

**Published:** 2026-08-28

**Authors:** Georgiy A. Demchenko, Alexandr E. Gulyayev, Sayagul A. Kairgeldina, Madina B. Baurzhan, Marat R. Khanturin, Kanat K. Tekebayev, Nazym S. Sagandykova, Laura U. Koibasova, Makpal A. Yessenova

**Affiliations:** 1 RSE on the Right of Economic Management Scientific Research Institute of Balneology and Medical Rehabilitation of the Ministry of Health of the Republic of Kazakhstan, Astana 010000, Kazakhstan; georgiidemchenko@mail.ru (G.A.D.); akin@mail.ru (A.E.G.); sanborovoe@mail.kz (S.A.K.); madina_baurzhan@mail.ru (M.B.B.); kanat_7@mail.ru (K.K.T.); doctor.ent.alm@gmail.com (N.S.S.); 2 Laboratory of Physiology Lymphatic System, Institute of Genetics and Physiology Sience Committee of the Ministry of Science and Higher Education of the Republic of Kazakhstan, Almaty 050060, Kazakhstan; khanturin55@gmail.com (M.R.K.); esenova_makpal@mail.ru (M.A.Y.); 3 National Laboratory Astana, Nazarbayev University, Astana 010000, Kazakhstan; 4 Department of Glinical Pharmacology and Evidence-Based Medicine, NC JSC “Karaganda Medical University”, Karaganda 100030, Kazakhstan

**Keywords:** bronchopulmonary system, dust-related lung diseases, pneumoconiosis, medicinal plants, herbal preparations, pulmonary rehabilitation, lymphatic sanitation

## Abstract

The aim of this study was to summarize current evidence on dust-related diseases of the bronchopulmonary system, medicinal plants, herbal formulations, and their underlying mechanisms of action. A literature review was conducted based on publications indexed in ScienceDirect, Google Scholar, SciFinder, Scopus, and Medline (PubMed), focusing on the mechanisms underlying dust-related diseases of the bronchopulmonary system, pneumoconiosis, and the potential of phytotherapeutic approaches to modulate these processes. The following keywords were used: bronchopulmonary system, pneumoconiosis, dust-related lung diseases, medicinal plants, herbal preparations, pulmonary rehabilitation, and lymphatic sanitation. The pathogenesis of pneumoconiosis involves the deposition of inhaled dust particles in the lung tissue, followed by chronic inflammation, oxidative stress, and progressive fibrosis. In experimental models, flavonoids, alkaloids, terpenoids, glycosides, tannins, and other phytochemical groups have demonstrated therapeutic potential in bronchopulmonary diseases. Among the compounds investigated, emodin, celastrol, kaempferol, sodium tanshinone IIA sulfonate, astragaloside IV, and dioscin have shown promising effects by modulating key signaling pathways and reducing inflammatory responses. Experimental evidence indicates that these phytochemicals can target major pathways involved in inflammation and fibrogenesis, thereby exerting antioxidant, anti-inflammatory, and antifibrotic effects. The lymphatic system also contributes to the pathogenesis of pneumoconiosis by transporting inhaled dust particles to regional lymph nodes, where their accumulation may promote lymph node sclerosis. Therefore, lymphatic sanitation may represent an important therapeutic mechanism of action of herbal formulations in pneumoconiosis. Our proposed lymphotropic herbal formulation enhances physiological lymphatic drainage and may positively influence the motility and functional activity of the tracheobronchial lymph nodes. The therapeutic potential of medicinal plants and herbal formulations in pneumoconiosis is supported by experimental findings for *Tripterygium wilfordii* (celastrol, through suppression of the *EDNRB/Kng1*–(endothelin receptor type B/kininogen1), *Delphinium*, *Camellia*, and *Berberis* species (kaempferol, through inhibition of TLR4 (Toll-like receptor 4 signaling), *Astragalus membranaceus* (astragaloside IV, through antifibrotic activity mediated by inhibition of the TGF-β1 (transforming growth factor beta 1)/Smad3 (SMAD family member 3) pathway), and *Reynoutria japonica* Houtt., *Rheum*, and *Aloe* species (emodin, through inhibition of Smad3 and NF-κB (nuclear factor kappa B) phosphorylation and reduction of TGF-β1, α-SMA (alpha-smooth muscle actin), collagen I, TNF-α (tumor necrosis factor alpha), and IL-1β (interleukin-1 beta) levels in lung tissue). For these and several other medicinal plants and their flavonoids, antifibrotic activity has been demonstrated through inhibition of the Akt/NF-κB (protein kinase B/nuclear factor kappa B) signaling pathway. A promising direction for future research is to evaluate the combined use of herbal formulations with established antifibrotic agents to determine whether synergistic therapeutic effects can be achieved.

## 1. Introduction

Occupational pneumoconioses are chronic respiratory diseases that develop as a result of prolonged inhalation of inorganic dust in occupational settings. They remain a significant issue in occupational medicine, particularly among workers exposed to mineral, metallic, and other types of industrial dust. Prolonged exposure to dust particles can lead to chronic inflammation, oxidative stress, tissue damage, and progressive fibrotic remodeling of the lungs, ultimately resulting in impaired respiratory function and a reduced quality of life [1].

Current treatment for pneumoconiosis primarily focuses on alleviating symptoms, improving respiratory function, preventing complications, and slowing disease progression. However, the irreversible nature of established fibrotic changes significantly limits therapeutic options, and there is currently a lack of effective treatments capable of directly altering the course of the disease. This highlights the need to explore additional therapeutic approaches that target the molecular and cellular mechanisms of lung injury caused by dust exposure.

In recent years, increasing attention has been paid to medicinal plants and their bioactive compounds as potential sources of antioxidant, anti-inflammatory, cytoprotective, and antifibrotic agents [2]. Experimental studies demonstrate that phytochemical compounds—such as flavonoids, alkaloids, terpenoids, and glycosides—can modulate signaling pathways involved in the development of oxidative stress, inflammation, and fibrogenesis, including NF-κB, MAPK, TGF-β1/Smad3, and Nrf2 [3]. Of particular interest is their effect on macrophage-mediated inflammatory responses, given the central role macrophages play in the development and progression of lung injury following dust exposure [4].

Another potentially significant aspect of phytotherapy is its possible influence on the lymphatic system. Pulmonary lymphatic vessels participate in maintaining interstitial homeostasis and transporting fluid, macromolecules, and cellular components from lung tissue to lymph nodes [5].

Upon exposure to occupational dust, impaired lymphatic drainage may contribute to the persistence of inflammatory processes [6].

In this regard, the potential impact of medicinal plants and herbal preparations on lymphatic function is of interest as an additional avenue of research; however, the clinical significance of such effects in pneumoconiosis requires further validation.

Despite the growing body of experimental data, the existing literature remains heterogeneous, and a significant portion of the results has been obtained from experimental models rather than clinical studies involving patients with occupational pneumoconiosis. Therefore, the therapeutic potential of medicinal plants must be evaluated taking into account the existing level of evidence.

The aim of this review is to summarize current data on the mechanisms of pneumoconiosis development and the therapeutic potential of medicinal plants and the biologically active compounds they contain, in the context of occupational diseases.

To ensure a comprehensive and up-to-date integration of scientific literature, a structured literature search was conducted across PubMed/MEDLINE, Scopus, Web of Science, and Google Scholar databases for peer-reviewed studies published up to 2026. The search included terms related to pneumoconiosis, occupational dust exposure, dust-induced lung diseases, medicinal plants, phytotherapy, phytochemicals, inflammation, oxidative stress, and fibrosis. Studies addressing occupational dust-related respiratory diseases and the protective or therapeutic effects of medicinal plants and plant-derived compounds were included. Duplicate records and publications unrelated to the objectives of the review were excluded. Titles and abstracts were screened, followed by full-text assessment of relevant publications. A total of 105 publications were included in the final narrative synthesis.

## 2. Pathophysiological Mechanisms of the Development of Bronchopulmonary System Diseases

According to the World Health Organization (WHO) global study “Socioeconomic Impact of Respiratory Diseases,” COPD affects 65 million people worldwide, with an annual mortality rate of 3 million.

The distribution of dust particles entering the respiratory tract depends on the particle size. Particles larger than 10 µm are retained in the nasal passages and eliminated, but with increased and deep breathing, 10–20% of particles enter the large airways. Particles 5–10 µm in size primarily deposit on the ciliated epithelium of the bronchi. Particle deposition increases sharply with shortness of breath, deep breathing, and bronchial inflammation. The final barrier to dust is the alveoli, where particles 0.5–5 µm in size penetrate. These particles are the ones that cause damage to the lung parenchyma. Dust is normally removed from the ciliated epithelium of the bronchi within 1 h. Particles deposited in the alveoli are engulfed by alveolar macrophages. After several hours or days, dust-laden macrophages migrate to the small airways containing the ciliated epithelium. Some particles, penetrating the respiratory epithelium, are engulfed by interstitial macrophages. Engulfed particles can remain in the interstitial tissue of the lungs for a long time; some are transported to regional lymph nodes, while others return to the respiratory tract and are eliminated [7].

Pneumoconiosis affects 26.6% to 53% of workers employed in mining, metallurgy, and other industries where large amounts of dust are present in the air [8]. A recent nationwide job-exposure matrix study conducted in Kazakhstan demonstrated that up to 53% of the working population is exposed to occupational vapors, gases, dust, or fumes, emphasizing the substantial occupational burden of hazardous aerosol exposure [9]. Pneumoconiosis is caused by prolonged inhalation of inorganic dust. When dust enters the respiratory tract, it partially settles on the mucous membranes, partially accumulates in the alveoli, and partially penetrates the lymphatic vessels and pleura. Residing on the mucous membranes, dust irritates receptors and causes excessive activity of the secretory glands. This results in chronic inflammation, which leads to the development of chronic dust bronchitis, obstructive pulmonary emphysema, and fibrosis. These diseases are among the leading causes of disability, disability, and mortality worldwide [10].

Lung diseases, including chronic obstructive pulmonary disease (COPD), asthma, silicosis, and acute respiratory distress syndrome (ARDS), are common causes of morbidity and mortality worldwide. Dust-induced lung diseases are currently considered among the most common occupational diseases [11].

ARDS is based on diffuse inflammation of the lungs. This process is divided into three phases: exudative, proliferative, and fibrotic [12]. During the exudative phase, cytokines and other pro-inflammatory substances are released in response to inflammation, activating alveolar macrophages and circulating neutrophils. Activated neutrophils, in turn, attach to the endothelium of pulmonary capillaries and release the contents of their cytoplasmic granules (proteases and toxic oxygen metabolites). This damages the capillary endothelium and alveolar epithelium, disrupting the alveolar–capillary barrier. As a result, exudate penetrates into the lung parenchyma and alveolar airspace. Gas exchange is impaired, and hypoxia occurs. In some patients, the process progresses to the fibrotic phase. Fibrin accumulated in the lungs undergoes remodeling and can cause fibrosis [13,14].

**Figure 1:**
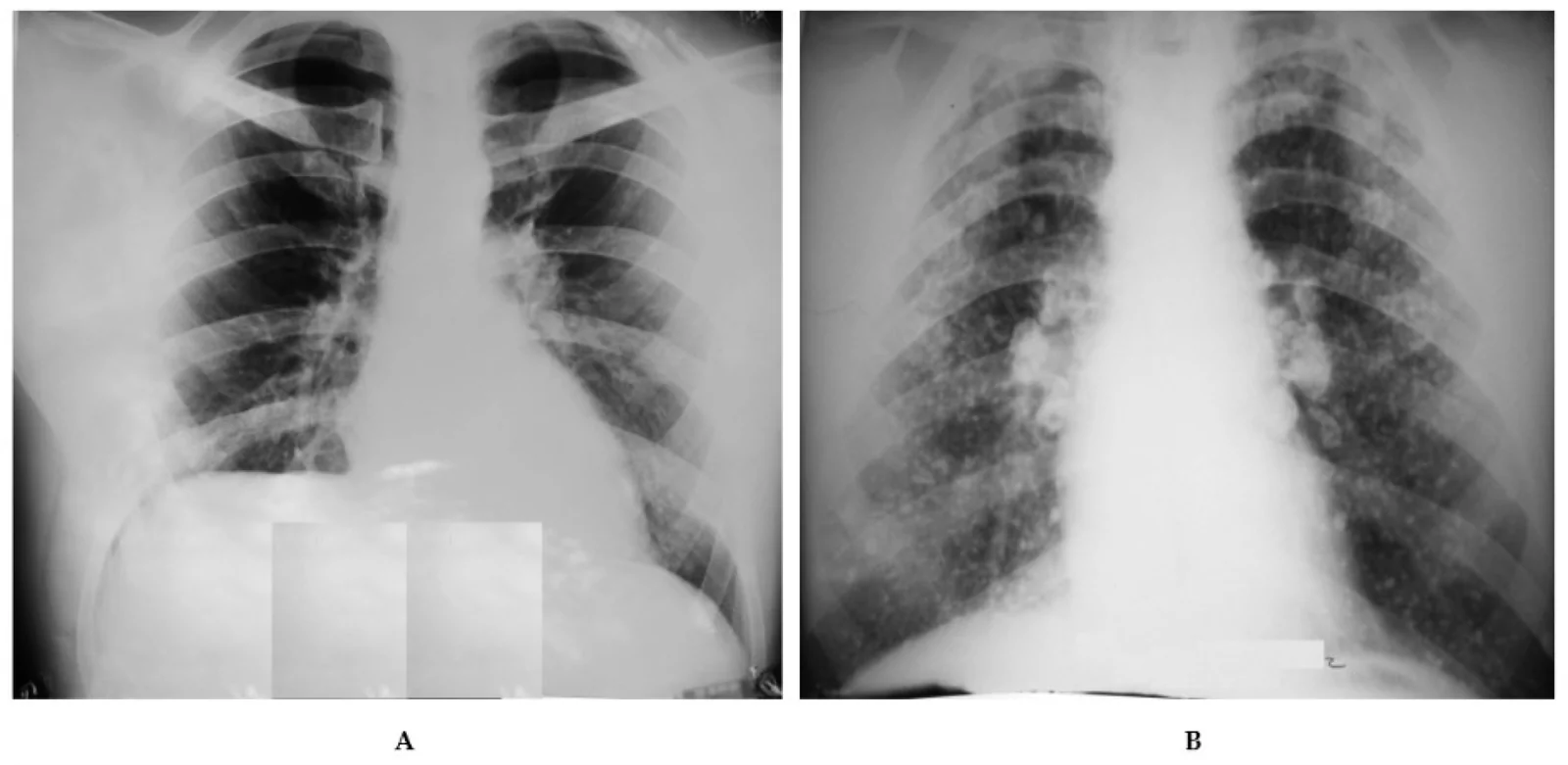
Chest X-rays: (**A**) normal; (**B**) silicosis with diffuse nodular and interstitial fibrous changes in the lung tissue.

As shown in Figure 1, the development of silicosis is accompanied by the appearance of diffuse, finely nodular changes in the lung tissue and deformation of the pulmonary pattern. The most pronounced changes are localized in the upper and middle sections of the lungs, Figure 1B, whereas on a normal chest radiograph (A), such changes are absent.

**Figure 2:**
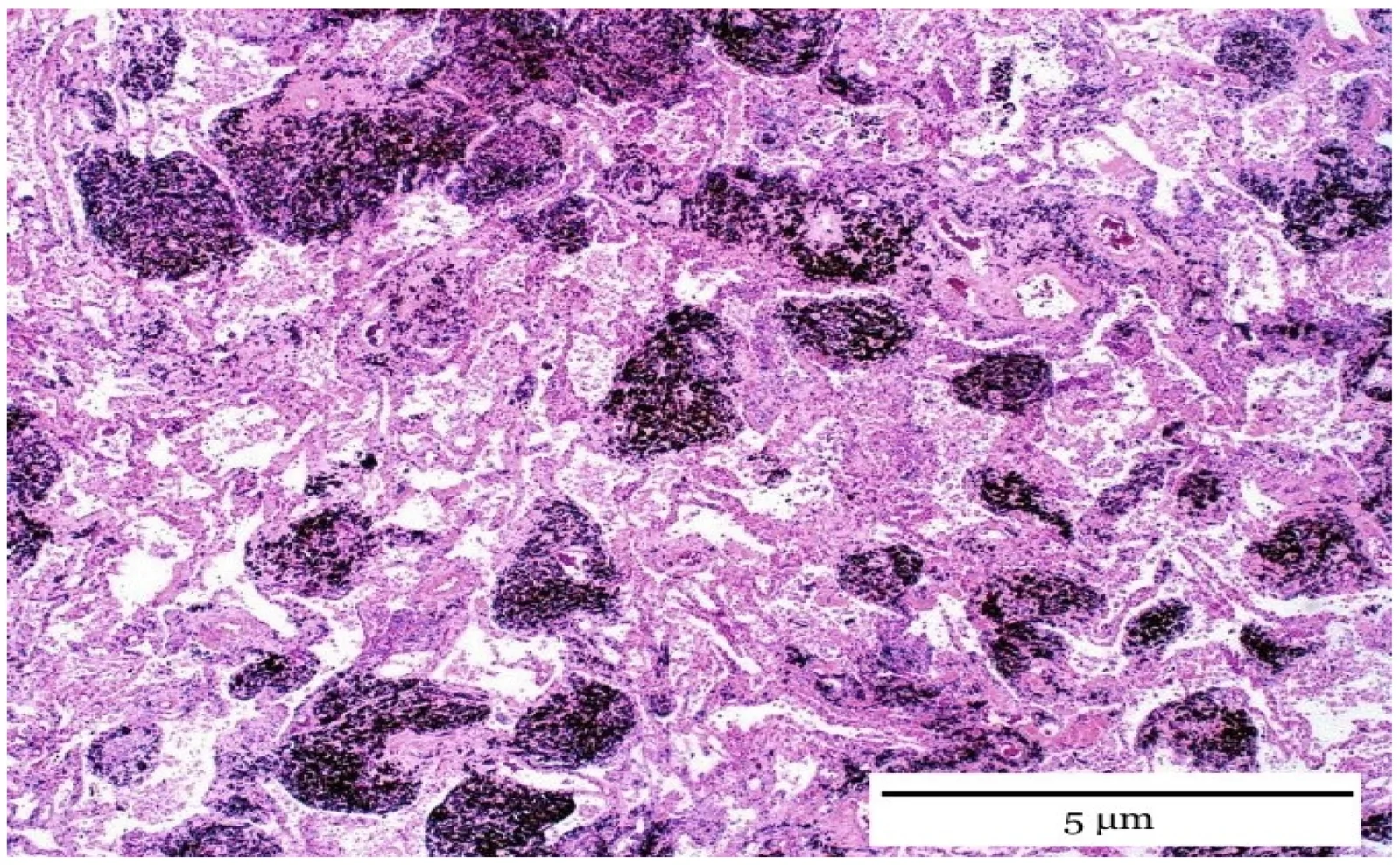
Microstructure of the lung in a patient with pneumoconiosis. Dark inclusions: dust particles in the tissue of the lung alveoli. Magnification 400×.

Many theories have existed regarding the pathogenesis of silicosis and other pneumoconioses. The most accepted theories were mechanical, chemical, biological, and piezoelectric. Currently, the immunological theory of pneumoconiosis suggests that silicosis is impossible without the phagocytosis of quartz particles by macrophages. Moreover, it is known that the rate of macrophage death is proportional to the fibrogenic aggressiveness of the dust. Macrophage death is the first and necessary step in the formation of a silicotic nodule. A necessary prerequisite for the development and formation of a nodule is the repeated phagocytosis of dust released from dying macrophages. The released pro-inflammatory mediators (cytokines and arachidonic acid metabolites) in turn induce the accumulation of inflammatory cells in the alveolar septa and epithelial space. Oxygen radicals lead to the destabilization and death of macrophages, damaging lung tissue. Proteolytic enzymes, such as metalloproteinases and elastase, released from damaged macrophages also destroy pulmonary structures. The inflammatory phase is accompanied by reparative processes, in which growth factors stimulate the production and proliferation of mesenchymal cells and regulate the formation of new vessels and epithelium in damaged tissues. Furthermore, fibrogenic dust particles independently activate pro-inflammatory cytokines. The role of tumor necrosis factor-alpha (TNF-alpha) and interleukin-1 in the development of silicosis has been established. Growth factors, particularly transforming growth factor-beta, play a key role in fibrogenesis. Its direct stimulating effect on fibroblast proliferation and the expression of collagen and fibronectin has been demonstrated (Figure 2) [15,16].

Manifestations of oxidative stress during the development of dust-related diseases (pneumoconiosis, bronchitis) include excessive formation of reactive oxygen species released by macrophages and leukocytes associated with inflammatory reactions in the lungs. These processes lead to apoptosis, extracellular matrix remodeling, alveolar epithelial damage, impaired mitochondrial respiration, membrane lipid peroxidation, increased mucus secretion, and oxidative inactivation of surfactants and antiproteases [17].

According to the generally accepted view, activated effector cells of the immune system are capable of synthesizing over 50 different mediators that promote inflammatory processes. This phenomenon, in turn, leads to bronchial obstruction, which is caused by edema, increased mucus secretion, changes in the rheological properties of sputum, and morphological restructuring of the bronchial tree. These processes can lead to the further development of pathogenic microflora in the bronchial tree. These processes may lead to impaired mucociliary clearance, resulting in the accumulation and proliferation of pathogenic microflora within the bronchial tree, which in turn triggers local activation of the immune response involving alveolar macrophages and inflammatory mediators.

It has been established that the mechanisms for clearing dust particles from the respiratory system are associated with the monocyte–macrophage system, which is considered the front line of immune defense. Along with immune and inflammatory mechanisms, mechanical airway clearance plays an important role in pulmonary defense, including mucociliary transport, the cough reflex, and the removal of particles via bronchial secretions. Impaired mucociliary function and changes in mucus properties contribute to particle retention, chronic inflammation, and progression of fibrosis. Several medicinal plants (e.g., thyme, primrose, ivy, plantain, verbena, and others) possess secretolytic and expectorant properties, improving mucociliary clearance and promoting airway cleansing. Alveolar macrophages participate in immune processes by phagocytosis of antigenic material by these cells, processing it, and delivering it in the appropriate immunogenic form to T and B lymphocytes. Activated proteases hydrolyze the connective tissue framework of the organ, leading to disruption of elastin and collagen, which then contribute to the development of fibrosis and emphysema [18]. In response to inhaled dust particles, airway and alveolar epithelial cells, macrophages, and other cells are known to produce various cytokines and growth factors, including macrophage inflammatory proteins (MIP1, MIP2), transforming growth factor-β (TGF-β), monocyte chemoattractant protein 1, and platelet-activating factor. These pro-inflammatory factors attract other immune cells to the lungs, which produce inflammatory mediators and cell damage, and activate fibroblasts, which increase the production of extracellular matrix proteins, leading to collagen deposition and ultimately lung scarring [19,20].

Coal dust has been shown to stimulate the release of cytokines important for pulmonary inflammation and fibrosis, including tumor necrosis factor-α (TNF-α) and IL-1 [21]. Compared with non-miners, miners with Coal workers’ pneumoconiosis (CWP) have elevated levels of cytokines in both serum and bronchoalveolar lavage, including IL-1β, IL-6, and TNF-α [22]. Furthermore, miners with chronic lung injury (CWP), with or without progressive massive fibrosis (PMF), have elevated levels of oxidative damage markers in bronchoalveolar lavage, including superoxide dismutase, glutathione peroxidase, and catalase, compared with controls. In a study of miners with early CWP, as detected by high-resolution chest computed tomography (CT), plasma antioxidant enzyme levels and activities (malondialdehyde, superoxide dismutase, and glutathione peroxidase) were elevated compared with controls [23], indicating the role of oxidative stress caused by increased free radical formation. Oxidative damage from coal dust exposure has also been suggested in experimental animal models of lung inflammation [24].

Despite shared core mechanisms in pneumoconiosis, including chronic inflammation, macrophage activation, oxidative stress, and fibrosis, different types of occupational dust exhibit specific pathogenetic features. Crystalline silica has the most pronounced fibrogenic potential, leading to silicosis. Silica particles are readily phagocytosed by alveolar macrophages, inducing their injury and death, followed by the release of pro-inflammatory cytokines, reactive oxygen species, and growth factors. Activation of the NLRP3 inflammasome and increased production of interleukin-1β play a key role in sustaining chronic inflammation and promoting fibrosis progression. Silicosis is characterized by the formation of typical silicotic nodules and the development of progressive massive pulmonary fibrosis.

Coal Workers’ Pneumoconiosis (CWP) develops due to prolonged exposure to coal dust containing carbon and varying amounts of silica. Unlike silicosis, coal dust has lower direct cytotoxicity; however, it promotes chronic macrophage activation and reactive oxygen species generation. The inflammatory process is characterized by accumulation of pigmented macrophages, formation of coal macules, and progression to interstitial fibrosis.

Asbestosis results from inhalation of asbestos fibers, which, due to their length and biopersistence, persist in lung tissue for long periods. Asbestos fibers induce oxidative stress, chronic inflammation, and fibroblast activation. A distinctive feature of asbestosis is predominant involvement of the lung interstitium and pleura, leading to diffuse interstitial fibrosis, pleural plaques, and an increased risk of malignant mesothelioma and lung cancer.

Besides mineral and organic occupational dust, heavy metals present in industrial aerosols may also make a significant contribution to the development of occupational respiratory diseases. Particular attention is given to exposure to lead (Pb), cadmium (Cd), chromium (Cr), nickel (Ni), and arsenic (As), which affect workers in mining, metallurgy, battery production, and other industrial sectors. Inhalation of lead-containing dust and fumes leads to damage of the alveolar epithelium, activation of inflammatory cells, and increased production of reactive oxygen species. The resulting oxidative stress is associated with activation of pro-inflammatory signaling pathways, including NF-κB, impairment of antioxidant defenses, and injury to lung tissue. In addition to local effects on the respiratory system, lead can cause systemic toxicity involving the nervous, cardiovascular, hematopoietic, and renal systems. Similar toxic mechanisms have been described for cadmium, chromium, and other heavy metals, which may further enhance inflammatory and fibrotic processes in the lungs.

Inhaled dust and heavy metals can cause inflammation and tissue damage in the lungs, which can disturb the normal balance of fluid in the lung tissue and increase the need for lymphatic drainage. Fluid, inflammatory substances, damaged cells, and dust particles can accumulate in the lung tissue and need to be removed through the lymphatic system. If lymphatic drainage is impaired or overloaded, these processes may contribute to prolonged inflammation and changes in lung tissue.

Lymphatic circulation and processes occurring in the lungs when dust particles enter. The lungs are characterized by a large volume of blood flowing through them. These circumstances require effective lymphatic drainage, which keeps the lungs dry and cleanses the pulmonary epithelium of small particles and toxic substances. Lymphatic vessels are involved in functions such as the control of interstitial fluid in the lungs and pleural space. Lymphatic vessels extend deep into the lobes of the lungs to the interalveolar septa [25].

**Figure 3:**
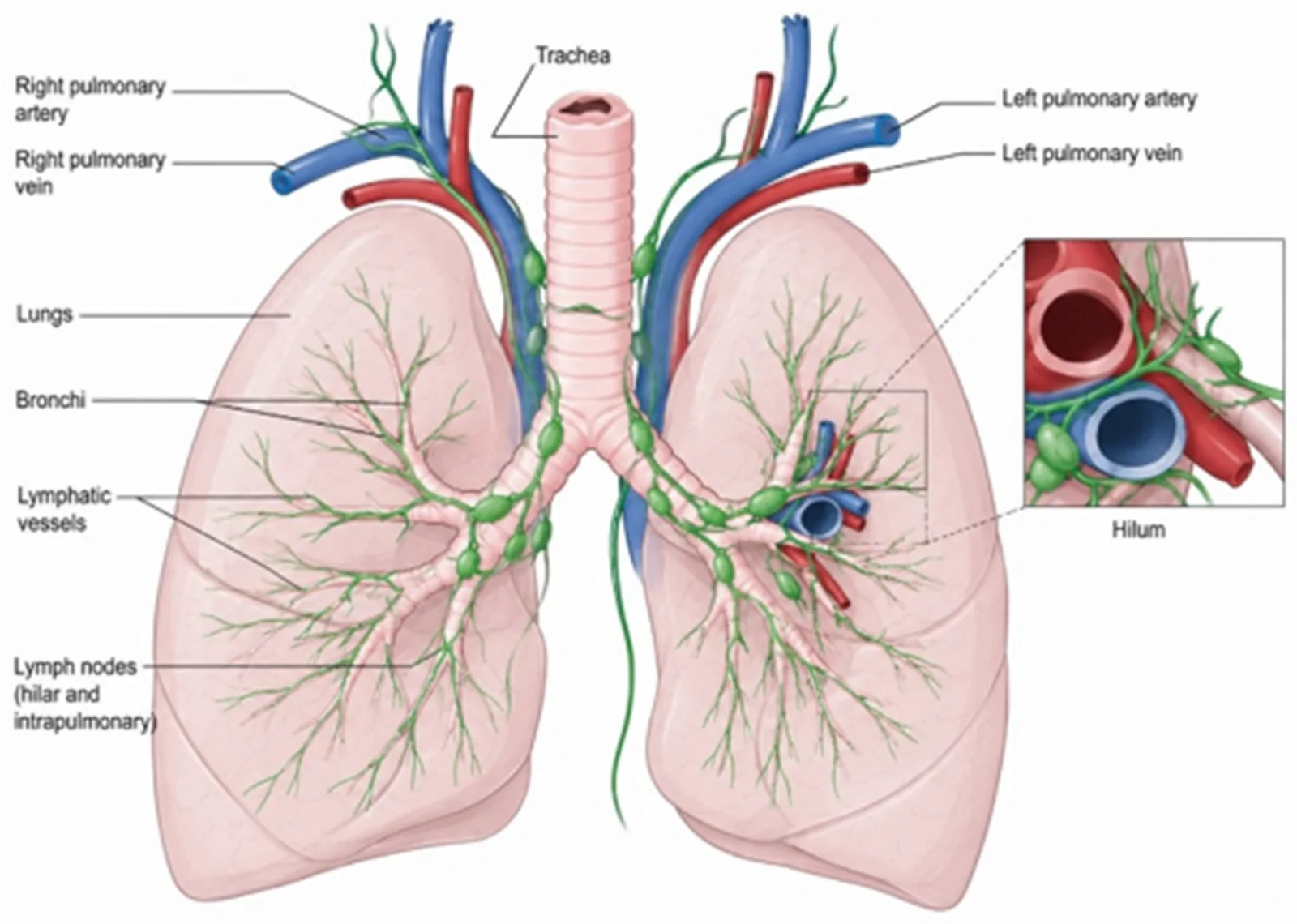
Lymphatic system of the lungs and respiratory tract.

Most lymphatic vessels in the lungs are located in the connective tissue surrounding the airways and blood vessels. Under physiological conditions, the lungs contain very little interstitial fluid: low tissue elasticity, low filtration pressure in the blood capillaries, low microvascular permeability, and a powerful lymphatic pump that promotes lymph formation and lymph drainage even under subatmospheric pressure keep the pulmonary interstitium virtually dehydrated to optimize the thickness of the air–blood barrier and gas diffusion [26,27]. Figure 3 illustrates the organization of the pulmonary lymphatic system, which is responsible for draining interstitial fluid and removing dust particles and toxic substances.

Lymph nodes are peripheral organs of the immune system, located along the lymphatic drainage pathway from organs and body parts. Beneath the lymph node capsule lies the subcapsular sinus, a crucial structure that determines the early response of the lymphatic system to an incoming antigen. Antigens, cytokines, and other molecules transported by the lymph interact with these cells, thereby triggering immune processes in the lymph node. Trabeculae, which act as supporting structures for the lymph nodes, extend from the capsule into the node. The space between the trabeculae contains lymphoid tissue organized into follicles. B-lymphocyte proliferation and differentiation occur in the area adjacent to the capsule. Below, in the paracortical zone, T-lymphocytes predominate, undergoing antigen-dependent proliferation and differentiation here. The lymph node performs several important functions, including the functions of a mechanical and biological filter. Bacteria, viruses, and cancer cells are retained in the reticular structure of the lymph node and analyzed by antigen-presenting cells [28]. Lymph nodes perform barrier-filtration, cytopoietic, and immunopoietic functions. In the lymph nodes, which are located along the lymphatic vessels and serve as biological and mechanical filters for the lymph flowing through them, the lymph undergoes detoxification, which is carried out at three levels: (1) biophysical processes (absorption, filtration, etc., in the reticular tissue); (2) biochemical transformation (lysozyme, complement system, tumor necrosis factor, enzymes-monoamine oxidases, cytochrome P-450, etc.); and (3) immunobiological processing (cellular and humoral immune response).

Effective lymphatic drainage during inflammation ensures timely removal of inflammatory mediators from the lung tissue. Deposition of particulate matter and the associated responses within lymphoid structures and lymphatic vessels vary significantly depending on the type of dust. Coal and silicon carbide particles generally do not persist within intrapulmonary lymphoid foci; instead, they predominantly accumulate in regional lymph nodes. In contrast, silica particles tend to accumulate within or in close proximity to lymphoid tissue foci. These particles induce progressive fibrotic changes and gradual obliteration of lymphatic structures, resulting in impaired or arrested lymph flow.

Particle size is a critical determinant of the severity of pulmonary injury following dust exposure. Intratracheal instillation of finely dispersed suspensions of coal dust, silica, iron compounds, or antimony produces an acute inflammatory response in lung tissue. This reaction is non-specific in nature, with the exception of the presence of exogenous particulate material. The extent of lung injury induced by silica increases as particle size decreases. Particles measuring 6–12 μm exert minimal biological effects compared with those in the 1–3 μm range. Particles of 0.6–1.0 μm demonstrate markedly higher biological activity, whereas particles around 3 μm produce little or no detectable effect [29].

The smallest particles (~0.26 μm) readily reach the alveoli, where they are retained and induce pronounced structural alterations. This process is accompanied by the accumulation of mononuclear cells and reticular elements within the alveolar spaces and interalveolar septa. Initially, discrete foci of injury are formed, which progressively coalesce, leading to extensive involvement of the pulmonary parenchyma. The bronchial and hilar lymph nodes are also affected and may increase in size up to threefold compared with normal [30]. Microparticulate dust (~0.6 μm) crosses the respiratory epithelium and is taken up by interstitial macrophages after bypassing mucociliary clearance mechanisms. These particles persist for prolonged periods within the interstitial lung tissue. A fraction is transported to regional lymph nodes, where it is retained, while only a minor portion reaches major lymphatic channels and subsequently enters the systemic circulation [30,31]. The lymphatic system plays a central role in the pathogenesis and progression of pneumoconiosis, contributing to the development of diffuse interstitial fibrosis.

Study of the pulmonary lymphatic system may contribute to a better understanding of the development of bronchopulmonary diseases, as the lymphatic system, by draining the interstitial space, contributes to the clearance of dust particles and pro-inflammatory mediators. This may provide a basis for further investigation of these pathologies, experimental modeling, and subsequent clinical studies.

## 3. Current Pharmacological Therapy for Pneumoconiosis

Pharmacological therapy for chronic dust-induced bronchitis should primarily aim to restore airway patency and correct the molecular and cellular disturbances underlying disease development [2]. The authors proposed the following therapeutic approaches to improve airway patency:

− Enhancement of the body’s antioxidant defense system to neutralize reactive oxygen species generated by coniophages and lipid peroxidation products that damage cellular membranes, using antioxidant and antiperooxidant agents;
− Increasing the resistance of alveolar macrophages, which are responsible for clearing deposited dust particles from the lungs, by delaying the development of cellular energy deficiency and intracellular hypoxia with glutamic acid and its salts;
− Facilitating the clearance of viscous bronchial secretions through the use of mucolytic and expectorant agents;
− Anti-inflammatory therapy with corticosteroids and antibiotics;
− Reducing respiratory failure and pulmonary hypertension through oxygen therapy and cardiovascular agents;
− Alleviating airway obstruction and improving respiratory tract airflow through the use of long-acting bronchodilators such as traventol [31];
− Promoting the synthesis of elastic fibers and restoration of the elastic framework of the lungs by restoring the balance between proteases and antiproteases through inhalation of naturally derived antiprotease preparations [32].

Despite the broad range of symptomatic and supportive treatment options, no effective pathogenetic therapy for pneumoconiosis is currently available. The major challenge is the irreversible nature of the fibrotic changes in lung tissue that develop following prolonged exposure to mineral dust. Most available therapeutic approaches are aimed at reducing symptom severity, improving respiratory function, and preventing complications, but they do not completely halt disease progression [33].

In routine clinical practice, patients generally receive symptomatic treatment with bronchodilators and mucolytic agents. Avoidance of further exposure to silica and other occupational dusts, as well as prompt treatment of respiratory infections, is also essential. Some improvement in pulmonary fibrosis, including silicosis, may potentially be achieved with agents that increase intracellular cyclic adenosine monophosphate (cAMP) or cyclic guanosine monophosphate (cGMP) levels, such as phosphodiesterase inhibitors, including roflumilast [34].

Phosphodiesterase inhibition increases intracellular cAMP levels and reduces the activation and dysfunction of leukocytes, bronchial and pulmonary vascular smooth muscle cells, endothelial cells, airway epithelial cells, and fibroblasts, as demonstrated in experimental models [35].

In vitro studies involving human neutrophils, monocytes, macrophages, and lymphocytes have shown that roflumilast and its active metabolite, roflumilast N-oxide, suppress the release of inflammatory mediators, including leukotriene B4, reactive oxygen species, tumor necrosis factor-α (TNF-α), interferon-γ, and granzyme B. In patients with COPD, roflumilast reduces neutrophil counts in sputum and decreases the recruitment of neutrophils and eosinophils into the airways in healthy volunteers exposed to endotoxin [36,37] or corticosteroids [38].

In recent years, considerable attention has been directed toward antifibrotic agents, particularly pirfenidone and nintedanib, which have demonstrated efficacy in idiopathic pulmonary fibrosis (IPF). Their potential use in progressive fibrosing interstitial lung diseases, including selected forms of pneumoconiosis, is currently being investigated. Pirfenidone exerts anti-inflammatory and antifibrotic effects by suppressing transforming growth factor-β (TGF-β) production and collagen synthesis, whereas nintedanib inhibits receptor tyrosine kinases involved in growth factor signaling and fibrogenesis. However, clinical evidence supporting the use of these agents in pneumoconiosis remains limited. Most available studies involve small patient populations, relatively short follow-up periods, and a lack of standardized efficacy endpoints. Treatment may also be associated with adverse events, including gastrointestinal disturbances, elevated liver enzyme levels, and reduced treatment tolerability. Further randomized clinical trials are therefore required to establish the efficacy, safety, and optimal treatment regimens of antifibrotic agents in pneumoconiosis [39].

More recently, nerandomilast (BI 1015550), an orally administered selective phosphodiesterase 4B (PDE4B) inhibitor, has been developed for the treatment of idiopathic pulmonary fibrosis and other progressive fibrosing lung diseases. The drug has anti-inflammatory, immunomodulatory, and potentially antifibrotic properties and is intended to slow the decline in lung function. Clinical studies have shown that nerandomilast may slow disease progression in patients with idiopathic pulmonary fibrosis. In patients with progressive pulmonary fibrosis, treatment with nerandomilast resulted in a smaller decline in forced vital capacity (FVC) than placebo over 52 weeks, supporting its potential as a therapeutic option for progressive fibrosing lung diseases [40].

## 4. Use of Herbal Preparations for the Treatment of Bronchopulmonary Diseases

Treatment of acute respiratory distress syndrome (ARDS) Acute lung inflammation is characterized by primary interstitial ARDS, represented by neutrophil infiltration. Sometimes, severe fibrosis is accompanied by edema and hypoxia, and, in addition, it is one of the leading causes of death in the intensive care unit [41]. Due to the complex pathophysiology, the treatment of ARDS is extremely challenging. However, recent studies conducted on experimental models have shown that there are several therapeutic approaches that can be beneficial. Among them, beneficial effects of plant-derived compounds that can influence various signaling pathways involved in the development of silicon-induced pulmonary fibrosis have been discovered [42]. However, the treatment of ARDS with herbal remedies has not yet been used clinically. There is considerable evidence that natural products and their combinations can help in the treatment of ARDS. In model experiments, flavonoids, alkaloids and glycosides are groups of phytochemicals that have shown their effectiveness for the treatment of ARDS [43].

Luteolin is a flavone found in celery, parsley, broccoli, and chamomile. In an experimental model of acute lung injury in animals, pretreatment with luteolin reduced pulmonary hemorrhage, neutrophilic inflammation, and interstitial edema.

Pretreatment with the flavonoid luteolin has been shown to reduce pulmonary hemorrhage, neutrophilic inflammation, and interstitial edema. The reduction in pulmonary inflammation was mediated by cytokines such as TNF-α and ICAM-1 in bronchoalveolar lavage fluid. Reduced catalase and superoxide dismutase activity regulates oxidative damage and lipid peroxidation. This is due to luteolin’s effects on NF-κB and mitogen-activated protein kinase (MAPK) signaling [35]. The mechanism of action of luteolin is associated with suppression of NF-κB-dependent transcription of pro-inflammatory mediators and modulation of MAPK signaling pathways, leading to reduced oxidative stress and inflammatory responses [44].

Quercetin is a flavonoid found in onions, apples, berries, tea, and some medicinal plants. In LPS-induced experiments, it reduces the release of pro-inflammatory cytokines in bronchoalveolar fluid (BALF) heme oxygenase-1 (HO-1) and affects the expression of TNF-alpha, IL1-β, and IL6 [45].

In animal studies of LPS-induced lung injury, quercetin reduced serum levels of TNF-α, IL-1β, IL-6, and nitric oxide (NO).

This process also leads to an increase in the secretion of IL10, an inflammatory cytokine pathway [42]. When tested in LPS-induced acute lung injury (ALI) models, quercetin has shown a reduction in pulmonary edema and hemorrhage, as well as alveolar surface thickness [41].

Quecetin reduced pulmonary inflammation and oxidative injury, partly through inhibition of NF-kB signaling, activation of HO-1, and suppression of pro-inflammatory cytokine and nitric oxide production [46].

COPD is characterized by persistent airflow limitation, usually progressive, and is associated with an exacerbation of the persistent inflammatory response to noxious substances or gases in the lungs and airways. This inflammatory response typically leads to parenchymal damage (emphysema), fibrosis, and damage to the small airways. The use of herbal remedies for the treatment of COPD is described in detail in a review article by J. Adamcakova and D. Mokra, 2021 [42].

Jaboticabine and 3,3′-dimethylella-4-O-sulfate are polyphenols isolated from *Myrciaria cauliflora*, also known as jaboticaba, a fruit native to Brazil. Jaboticaba extracts have been shown to be effective for the anti-inflammatory treatment of COPD [47]. Thus, they exhibit anti-inflammatory activity by suppressing inflammatory mediators and oxidative stress.

Celastrol is a widely studied anti-inflammatory traditional Chinese medicine extracted from *Tripterygium wilfordii*. It contains triterpenoid metabolites capable of suppressing the NF-kappaB (nuclear factor kappa B) signaling pathway. Through *EDNRB* gene expression, celastrol significantly reduces lung damage in lung diseases and is also involved in cellular apoptosis. Celastrol is excellent in suppressing *EDNRB/Kng1*(Endothelin receptor type B/kininogen 1) expression in cell and animal models and alleviates COPD through the inhibitory mechanism of the signaling pathway [48]. *EDNRB* (endothelin receptor type B) is involved in the regulation of vascular tone, inflammation, and tissue remodeling. Increased activity of the endothelin system may contribute to inflammatory infiltration and the development of pulmonary fibrosis. *Kng1* encodes kininogen-1, which is involved in inflammatory responses and tissue injury. Therefore, inhibition of the *EDNRB/Kng1* axis may limit inflammatory and fibrotic processes in lung tissue [48].

Sodium tanshinone sulfonate IIA (STS) is a water-soluble extract of Chinese sage (*Salvia miltiorrhiza*), which is used in traditional Chinese medicine for the treatment of coronary artery disease. Moreover, STS has demonstrated potent antioxidant, anti-inflammatory, and anti-apoptotic effects in various diseases by regulating various transcription factors, including nuclear factor kappa-B (NF-κB) (a key protein complex-transcription factor that controls the expression of genes involved in the immune response, inflammation, apoptosis, and cell proliferation), and by influencing Ca^2+^, K^+^, and Cl^−^ ion channels. In a rat model of silicosis, intraperitoneal administration of STS significantly reduced lung collagen levels and decreased ROS, as evidenced by a decrease in the production of malondialdehyde (MDA), a marker of lipid peroxidation under oxidative stress. The authors suggest that the antifibrotic effects of STS may be associated with increased expression of nuclear factor erythroid 2 (Nrf2) (a key transcription factor regulating cellular defense mechanisms against oxidative stress, toxins, and inflammation) and nuclear translocation, followed by an increase in the transcription of thioredoxin and its reductase [49]. In studies using a rat model of silicosis, STS demonstrated antioxidant and anti-inflammatory effects through activation of the Nrf2 pathway and inhibition of NF-κB, as well as reduction of oxidative stress [49].

Kaempferol (Kae) is a flavonoid widely distributed in various plant genera, including larkspur (*Delphinium*), camellia (*Camellia*), barberry (*Berberis*), citrus fruits, cabbage, onion, apple, and others. Kae exhibits broad anti-inflammatory, antioxidant, and antifibrotic effects, including inhibition of TLR4 receptors, NF-κB, and the MAPK pathways; suppression of IL-6, IL-1β, IL-18, and TNFα release; attenuation of oxidative stress through activation of the Nrf2-ARE signaling pathway; and more [48]. Since activation of the TLR4/NF-κB signaling pathway promotes the production of TNF-α, IL-1β, and other inflammatory mediators involved in the formation of silicotic nodules and the progression of pulmonary fibrosis, inhibition of these pathways by kaempferol may help reduce inflammation and slow the development of fibrotic changes [50].

In a mouse model of silicosis, intraperitoneal administration of Kae modulated silica-induced autophagy and significantly suppressed lung inflammation at day 7, as evidenced by lower expression of pro-inflammatory cytokines, and inhibited silica-induced pulmonary fibrosis at day 28, as evidenced by suppressed activation of matrix metalloproteinase-2 and smaller silicotic nodules [51].

Astragaloside IV (ASV) is one of the most active compounds isolated from the root of *Astragalus membranaceus*. This plant is widely used in traditional Chinese medicine for the treatment of several fibrotic diseases, including silicosis and pulmonary fibrosis [52]. The therapeutic effect of ASV may be mediated in part by suppressing TGF-β1/Smad3 signaling (a key mechanism of signaling from transforming growth factor-beta (TGF-β) receptors to the cell nucleus, where Smad3 acts as a transcription factor, regulating the expression of genes controlling proliferation, differentiation, fibrosis, and apoptosis). This pathway, as continuous phosphorylation of Smad3, is a major factor inducing fibrosis [53].

The antioxidant and anti-inflammatory effects of ASV may also be associated with the inhibition of NF-κB expression [50]. In an in vitro study, ASV prevented the invasion of TGF-β-induced cells, delaying the development of pulmonary fibrosis. Furthermore, ASV reduced the expression of the NLRP3 inflammasome (its activation leads to the assembly of a multimeric complex, activation of caspase-1, and the release of pro-inflammatory cytokines IL-1β and IL-18, causing inflammation) [51]. It exerts antifibrotic and anti-inflammatory effects through inhibition of the TGF-β1/Smad3 pathway, NF-κB signaling, and the NLRP3 inflammasome [54,55].

Dioscin is a widespread steroidal saponin present in a variety of vegetables and herbs, particularly in *Dioscoreaceae species* (*Dioscoréa caucásica*) [54]. In mouse models of pulmonary silicosis, daily oral administration of dioscin exerted anti-inflammatory and antifibrotic effects by reducing alveolar macrophage apoptosis and stimulating autophagy, a specific protective form of cell elimination. Dioscin-stimulated autophagy reduced silica-induced mitochondrial ROS release, mitochondrial dysfunction, and alveolar macrophage apoptosis, resulting in decreased production of pro-inflammatory factors, as well as decreased pulmonary inflammatory infiltration and collagen deposition [55].

Oral administration of dioscin to mice with silicosis reduced the influx of Fibrocytes, which are a direct source of fibroblasts, prevented fibroblast activation, and inhibited TGF-β/Smad3 signaling, which together resulted in decreased collagen deposition and slowed fibrosis. Furthermore, dioscin protected epithelial cells from silica-induced damage and reduced the infiltration and secretion of macrophages and lymphocytes in the lungs [56,57].

Oleanolic acid (OA) is a pentacyclic terpenoid of plant origin that is naturally found in vegetable oil, foods, and some medicinal herbs, either as the free acid or as the aglycone of triterpenoid saponins. OA has numerous pharmacological properties, including hepatoprotective, antioxidant, anti-inflammatory, and anticancer effects [58]. The anti-inflammatory and anticancer effects are likely mediated by actions on the NF-κB pathway [59].

Thus, OA and its derivatives have a wide range of potential applications in diseases associated with inflammation and oxidative stress, including pulmonary silicosis. Daily intragastric administration of OA to rats with silicosis significantly reduced the degree of silicon-induced fibrosis, including the content of collagen types I and III, and decreased oxidative stress, as reflected by a decrease in MDA content and an increase in the activity of antioxidant systems (superoxide dismutase and glutathione peroxidase) in the lungs. Furthermore, OA reduced the levels of TNF-α and TGF-β1. These results suggest that the protective effects of OA may be related to both its antioxidant activity and its ability to reduce cytokine expression and suppress collagen synthesis by modulating the Akt/NF-κB pathway [58]. It exerts antioxidant and anti-inflammatory effects through inhibition of NF-κB and modulation of the Akt/NF-κB pathway [58,59,60].

Hesperetin (HSP) is a flavonoid found primarily in citrus fruits that exhibits broad anti-inflammatory, antioxidant, antibacterial, and anticancer effects [61]. The anti-inflammatory effect of HSP is likely mediated by upregulation of peroxisome proliferator-activated receptor (PPAR)-γ and inhibition of NF-κB pathway activation [62]. In silica-fed rats, HSP reduced the severity of alveolitis and pulmonary fibrosis, decreased MDA levels, increased antioxidant enzyme activity and total antioxidant capacity, inhibited TGF-β1 synthesis and secretion, and decreased levels of pro-inflammatory cytokines IL-1β, IL-4, and TNFα [63]. The mechanism of action is associated with activation of PPAR-γ and inhibition of NF-κB signaling, which reduces inflammation and oxidative stress [61,62,63,64].

Emodin can be obtained from several plants, including *Reynoutria japonica* Houtt., *Rheum*, and *Áloë*. Emodin is used in traditional medicine in East and South Asia for its anti-inflammatory, anticancer, and antifibrotic effects [58]. The anti-inflammatory effects of emodin have been demonstrated in models of lipopolysaccharide-induced lung inflammation, which may be mediated by inactivation of the NF-κB pathway [65].

In mice administered bleomycin, emodin reduced biochemical markers (hydroxyproline content and myeloperoxidase activity) and histopathological markers of pulmonary fibrosis and also decreased the levels of TGF-β, IL-4, and IL-13 in BALF. Furthermore, emodin reduced fibroblast proliferation and collagen production in cell culture in a dose-dependent manner [66]. In a later study, emodin improved lung function and survival in bleomycin-challenged animals, reduced TNFα, IL-6, TGF-β1, and heat shock protein, decreased pulmonary edema, decreased collagen deposition, and α-SMA expression suppressed lung infiltration by myofibroblasts, macrophages, and lymphocytes and inhibited TGF-β1-induced Smad2/3 and STAT3 activation [67]. In pulmonary silicosis models, emodin reduced the extent of alveolitis and fibrosis in the lungs, inhibited Smad3 and NF-κB phosphorylation, and reduced the levels of TGF-β1, α-SMA, collagen-I, TNFα, and IL-1β in lung tissue. Furthermore, emodin inhibited apoptosis by suppressing the activity of the pro-apoptotic protein Bax and the activation of the anti-apoptotic protein Bcl-2. In addition, emodin reduced the expression of fibrosis, apoptosis, and inflammation markers in human macrophages cultured in vitro and alveolar epithelial cells [68]. The above results indicate that emodin can regulate the inflammatory response, EMT, and fibrosis process at multiple levels by inhibiting the TGF-β1/Smad3 signaling pathway and NF-κB signaling pathway. The proposed mechanism involves inhibition of the NF-κB and TGF-β1/Smad3 pathways, suppression of inflammatory cytokines, and attenuation of fibrotic processes [68].

**Figure 4:**
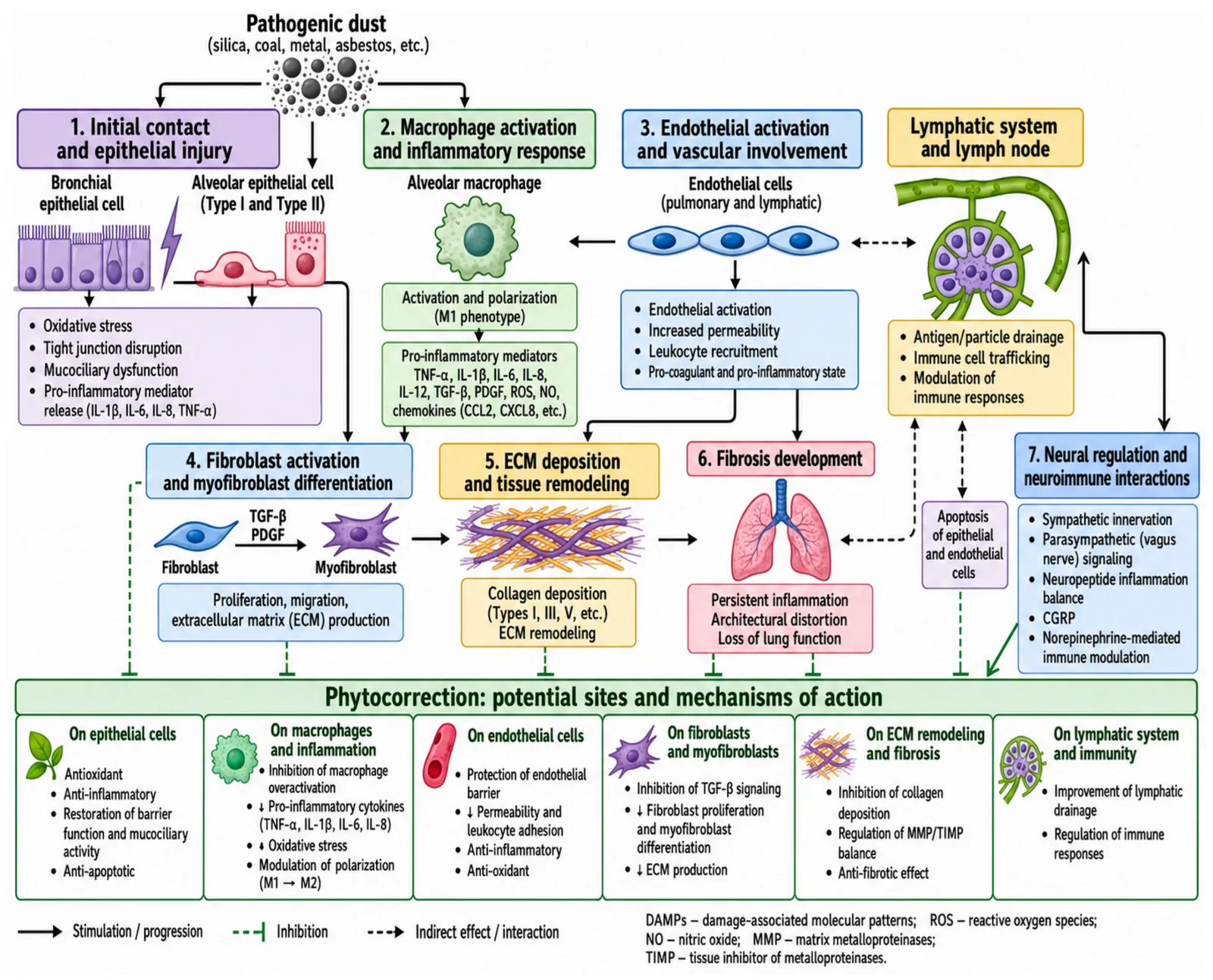
Stages of pneumoconiosis development and mechanisms of phytocorrection of the pathological process with herbal preparations. Dust particles induce epithelial injury, oxidative stress, macrophage and endothelial activation, inflammation, and lymphatic involvement, contributing to fibroblast activation, extracellular matrix remodeling, and fibrosis development. Neural and neuroimmune mechanisms may further modulate the inflammatory response. The lower panel illustrates the potential effects of phytocorrection, including antioxidant, anti-inflammatory, immunomodulatory, endothelial-protective, antifibrotic, and lymphatic-supportive effects. Solid arrows indicate stimulation or disease progression, whereas dashed lines indicate inhibition or indirect interactions.

Figure 4 presents a schematic illustrating the main stages in the development of the pathological process in pneumoconiosis and the mechanisms of phytocorrection at these stages using plant-derived preparations. Phytochemical compounds, including emodin, celastrol, kaempferol, sodium tanshinone IIA sulfonate, astragaloside IV, dioscin, and others, can modulate key signaling pathways involved in inflammation and fibrogenesis, including NF-κB, IL-1β, IL-4, IL-6, TNF-α, and TGF-β1/Smad3, thereby exerting antioxidant, anti-inflammatory, and antifibrotic effects.

## 5. Medicinal Plants with Therapeutic Potential for the Prevention, Treatment, and Rehabilitation of Dust-Related Bronchopulmonary Diseases

Herbal preparations have been widely used in traditional and complementary medicine, and their potential role in the management of various diseases continues to be investigated [69].

Currently, herbal medicine is increasingly being introduced into medical practice not only for the prevention but also for the treatment of many diseases. People strive to use synthetic drugs as little as possible, choosing natural herbs and infusions as an alternative. Plants contain a diverse range of natural chemical compounds, allowing for the development of treatment programs that take into account all the causes and mechanisms of their development [70]. Herbal medicines must meet the same quality, efficacy, and safety criteria as chemical drugs. Unlike synthetic drugs, they offer a broader spectrum of efficacy, fewer side effects, and lower risk of interactions with other medications. Phytotherapy is the most physiological method, significantly less likely to cause allergic and adverse reactions. A rational combination of medicinal plants, as well as their combination with synthetic drugs, can achieve the desired therapeutic effect, achieve sustained remission, and improve the patient’s quality of life (Table 1).

Table 1 presents medicinal plants with therapeutic potential for bronchopulmonary diseases, including pneumoconiosis. These plants are distributed across different continents and have been used in both conventional and traditional medicine. The table summarizes their major bioactive constituents, including polyphenols, vitamins, glycosides, flavonoids, organic acids, and other compounds, as well as the molecular mechanisms underlying their biological effects. The data presented in the table may provide a useful basis for further research into the prevention and treatment of bronchopulmonary diseases, including pneumoconiosis.

## 6. Phytocompositions for the Prevention and Treatment of Bronchopulmonary Diseases

The scientific literature cites a large number of medicinal plants that have a positive effect in the treatment of bronchopulmonary diseases, including bronchodilators, expectorants, mucus reducers, and anti-inflammatory, antioxidant, and cytoprotective properties. Since no single medicinal plant can provide the full spectrum of properties for treating bronchopulmonary pathologies, we recommend using phytocompositions.

Bronchipret (thyme and ivy leaf extract) has antitussive, expectorant, and anti-inflammatory effects. A 10-day course of treatment with appropriate doses of the drug resulted in a significant reduction in symptom severity or recovery, with excellent tolerability. Thyme contains essential oils (carvacrol and thymol), which promote sputum removal and clear mucus from the lungs; flavonoids, which have a bronchodilatory effect; and thymol, which, in addition to its mucolytic effect, also has antiseptic properties [100].

Thyme extract and primrose root have different pharmacological properties, which, when combined, work synergistically, providing expectorant, bronchospasmolytic, secretolytic, and anti-inflammatory effects. Thus, the bioactive compounds of primrose root are saponins and phenolic glycosides (primeverin). Saponins exert an expectorant effect by stimulating the upper gastrointestinal tract, which reflexively induces bronchial secretions [82]. In the scientific literature we have come across the following: Collection 1 contains narrow-leaved fireweed, bird’s knotweed, bogbean, Bidens and pennycress in the following component ratio 1:1:1:1: 1. Collection 2 contains narrow-leaved fireweed, marsh rosemary, birch, plantain, stinging nettle, calla lily (root), and bogbean in the following component ratio 1:1:1:1:1:1:1:1. Collection 3 contains narrow-leaved fireweed, field horsetail, stinging nettle, calla lily (root), cassandra, may-flower or lily of the valley, coltsfoot, and meadow vetch in the following component ratio of 1:1:1:1:1:1:1:1. When taken, the proposed herbal infusions improve overall health, activate the immune system, and provide a comprehensive effect on the body, producing a pronounced therapeutic effect in the treatment of upper respiratory tract infections [101,102].

The cytoprotective effects of polyphenolic extracts of cranberries, blueberries, and lingonberries have been established in vitro in cell cultures. The polyphenolic composition of cranberry, blueberry, and lingonberry extracts from the northwestern region of Western Siberia was determined, with the highest concentration of polyphenolic components found in the blueberry extract. Cytoprotective potential, dependent on polyphenol concentration, was demonstrated for all northern berry extracts in vitro experiments [98]. We have proposed a new lymphotropic phytocomposition consisting of Balsam Vozrozhdenie, *Ziziphora bungeana*, *Hawthorn almaatensis*, St. John’s wort, *Bergenia crassifolia*, *Hedysarum*, and *Echinacea purpurea*. It exhibits immunoprotective, antioxidant, anti-inflammatory, and immunotropic effects on age-related changes in the composition of biological fluids (blood, lymph, interstitial fluid) in Sprague-Dawley white laboratory rats. Administration of the phytocomposition for 3 months resulted in an 8% increase in interstitial fluid (IF) and plasma volume, a 31% increase in lymph flow, and a 41% increase in diuresis. Lipid levels in the blood and lymph decreased by 12%. An increase in the number of erythrocytes, leukocytes, immunoglobulins (except IgG), and lymphocyte subpopulations was also noted, which contributed to the strengthening of the immune properties of the blood and lymph. Increased tissue hydration and the antiatherogenic properties of lymph and blood lead to the restoration of the drainage and detoxification function of the lymphatic system. Thanks to bioflavonoids, microelements, and vitamins, the phytocomposition has a lymphotropic effect, changing the composition of blood, lymph, and interstitial fluid, stimulates the movement of fluid from the interstitium into the vascular bed, enhancing natural lymphodetoxification, and improves the immune properties of the blood and lymph. This phytocomposition also has anti-inflammatory and antioxidant effects, all of which suggest a positive effect on the bronchopulmonary system [102,103,104].

## 7. Conclusions

Pneumoconiosis has a complex pathophysiology, which makes its treatment difficult. Clinical studies and experimental models of dust-related bronchopulmonary diseases have shown beneficial effects of herbal preparations. These preparations can act on different signaling pathways and may contribute to therapeutic and rehabilitation effects. The main groups of phytochemicals with potential therapeutic activity in pneumoconiosis include flavonoids, alkaloids, terpenoids, and glycosides.

For example, in the treatment of acute respiratory distress syndrome (ARDS), luteolin and quercetin have shown beneficial effects by reducing pulmonary hemorrhage, neutrophilic inflammation, and interstitial lung edema. Several compounds, including Emodin, Celastrol, Kaempferol, Sodium Tanshinone IIA Sulfonate, Astragaloside IV, and Dioscin, have also demonstrated beneficial effects in COPD by affecting different signaling pathways and reducing inflammatory responses. Their anti-inflammatory effects are generally associated with a reduction in excessive reactive oxygen species production, inhibition of pro-inflammatory mediators through suppression of NF-κB translocation and mitogen-activated protein kinase (MAPK) phosphorylation, inhibition of TGF-β1/Smad3 signaling involved in fibrosis, and activation of nuclear factor erythroid 2-related factor 2 (Nrf2).

Particular attention should be given to medicinal plants containing flavonoids with antifibrotic properties, including *Tripterygium wilfordii*, *Delphinium*, *Camellia*, *Berberis*, *Astragalus membranaceus*, *Reynoutria japonica* Houtt., *Rheum*, and *Aloe.* In the treatment of pneumoconiosis, lymphatic clearance mechanisms may also be important, as they may contribute to reducing pathological changes during phytotherapy. The proposed lymphotropic phytocomposition stimulates the movement of fluid from the interstitial space into the vascular system, enhances the contractile activity of lymph nodes, supports natural lymphatic clearance, and improves the immune properties of blood and lymph.

This phytocomposition also has anti-inflammatory and antioxidant effects, which may contribute to its beneficial effects on the bronchopulmonary system. Several medicinal plants and herbal combinations show therapeutic potential for pneumoconiosis. For example, *Tripterygium wilfordii* (celastrol) may act through suppression of the *EDNRB/KNG1* axis. *Delphinium*, *Camellia, and Berberis* contain kaempferol, which can inhibit TLR4 receptors. *Astragalus membranaceus* contains astragaloside IV, which has antifibrotic effects through inhibition of the TGF-β1/Smad3 pathway. *Reynoutria japonica* Houtt., *Rheum*, and *Aloe* contain emodin, which has been reported to inhibit Smad3 and NF-κB phosphorylation and reduce the levels of TGF-β1, α-SMA, collagen I, TNF-α, and IL-1β in lung tissue. Other medicinal plants and their flavonoids have also demonstrated antifibrotic activity, including through inhibition of the Akt/NF-κB pathway.

A promising direction for future research is to evaluate the combined use of phytocomplexes with antifibrotic drugs to determine whether they may produce a synergistic effect. Further clinical studies are needed to confirm the efficacy and safety of these approaches in patients with pneumoconiosis.

## 8. Future Research Directions

Despite encouraging experimental findings, the clinical application of phytochemicals in dust-induced lung diseases remains insufficiently explored. Therefore, it is necessary to identify the most promising plant-derived compounds with pronounced anti-inflammatory and antifibrotic properties, particularly quercetin, luteolin, as well as extracts of Astragalus, sage, and turmeric. Further studies should focus on the standardization of herbal preparations, determination of effective dosages, and investigation of their bioavailability and pulmonary tissue distribution. Clinical trials should include clearly defined endpoints, such as pulmonary function tests, exercise capacity, respiratory symptoms, and assessment of pulmonary fibrosis using high-resolution computed tomography (HRCT). Standardized dosing regimens should also be established to improve reproducibility and facilitate comparison between studies. Improvement of experimental models that closely replicate chronic dust exposure conditions is also required. Another important direction is the evaluation of combined use of phytochemicals with antifibrotic drugs to assess potential synergistic effects. Ultimately, well-designed clinical trials are needed to confirm the efficacy and safety of these approaches in patients with pneumoconiosis.

## Figures and Tables

**Table 1:** Plants with therapeutic potential for dust-induced bronchopulmonary diseases.

Species (Common Name) and Therapeutic Effects	Major Bioactive Compounds and Molecular Mechanisms of Action
*Aesculus hippocastanum (Horse Chestnut)* The flowers, fruits, and bark of horse chestnut contain tannins, aesculin, and aescin. Alcoholic extracts are mainly used for vascular disorders and may also exert anti-inflammatory effects, including in bronchitis and other respiratory conditions [71].	Aesculin reduces capillary permeability, increases the antithrombotic activity of blood serum, enhances antithrombin production, and improves venous blood flow. Aescin decreases blood viscosity. Aesculin inhibits NF-κB and MAPK signaling pathways and suppresses IL-1β, IL-6, and TNF-α production. Aescin reduces TNF-α release.
*Nigella sativa (Black Cumin)* Extracts and seed oil of *Nigella sativa* exhibit antiviral, antidiabetic, cardioprotective, antioxidant, and anti-inflammatory effects. Its bioactive compounds may help regulate inflammation, oxidative stress, immune responses, and autophagy [72].	Thymoquinone is the principal biologically active constituent. Thymoquinone activates Nrf2 (↑ catalase, SOD), inhibits NF-κB, and downregulates TNF-α, IL-1β, and IL-8.
*Dracocephalum rupestre* *Dracocephalum rupestre* is traditionally used for respiratory disorders. Its flavonoid eriodictyol exhibits antioxidant and anti-inflammatory effects and may alleviate acute lung injury by activating the Nrf2 pathway and suppressing inflammatory cytokines [73].	Thymoquinone is the principal biologically active constituent. Eriodictyol activates Nrf2, increasing glutathione and superoxide dismutase levels.
*Glycyrrhiza glabra (Licorice)* The roots and rhizomes of *Glycyrrhiza glabra* are traditionally used for respiratory disorders due to their expectorant, demulcent, and anti-inflammatory properties. Licorice has also been investigated for potential therapeutic and preventive effects against SARS-CoV-2 infection [74,75].	Eriodictyol: The abundance of hydroxyl (-OH) groups in its molecular structure enables efficient electron donation, thereby neutralizing reactive oxygen species (ROS) and preventing oxidative stress. Glycyrrhizic acid: The major active constituent (present mainly as salts). It exhibits pronounced anti-inflammatory, antiviral, and expectorant activities. Its mechanism of action resembles that of adrenal corticosteroid hormones. Glycyrrhetinic acid: possesses potent antihistamine and anti-inflammatory properties. Flavonoids: liquiritin, isoliquiritigenin, and glycycoumarin. These compounds protect cells from damage, alleviate smooth muscle spasms, and regulate gastric acidity. Saponins: Natural surfactant-like compounds that stimulate mucosal receptors, enhancing secretion and facilitating sputum clearance in bronchitis. Glycyrrhizic acid inhibits phospholipase A_2_, reducing arachidonic acid release and prostaglandin E_2_ synthesis. Glycyrrhetinic acid suppresses NF-κB-mediated inflammatory cascades. Liquiritin activates Nrf2 and inhibits NF-κB and MAPK pathways.
*Astragalus membranaceus* *Astragalus membranaceus* is traditionally used for respiratory disorders and asthma. Its immunomodulatory effects may reduce airway inflammation by regulating the Th1/Th2 cytokine balance [76].	Polysaccharides: Complex carbohydrates that stimulate immune responses, enhance interferon production, and protect against viral and bacterial infections. Saponins (Astragaloside): unique triterpenoid compounds that strengthen blood vessels, reduce low-density lipoprotein (“bad”) cholesterol levels, and support myocardial health. Flavonoids (including quercetin and kaempferol): plant-derived antioxidants that neutralize free radicals, protect cells from aging-related damage, and suppress inflammatory processes. Quercetin & kaempferol inhibit NF-κB, COX-1/COX-2, and reduce prostaglandin and leukotriene synthesis.
*Scutellaria* spp. (*Skullcap*) Wogonin from *Scutellaria* roots exhibits anti-inflammatory effects in bronchial asthma by inhibiting IL-4/STAT6 signaling and reducing eotaxin-3 expression [77].	Baicalin: The principal bioactive compound responsible for the pronounced sedative, anti-inflammatory, and antioxidant effects of Scutellaria species. Baicalein: a potent antioxidant with neuroprotective and antiviral properties. Wogonin: a flavonoid that enhances sedative and anxiolytic effects. Baicalin & baicalein suppress IL-6, TNF-α, and IL-1β production. Wogonin inhibits NF-κB and activates Nrf2-mediated antioxidant defenses. Scutellarein: promotes smooth muscle relaxation and contributes to blood pressure reduction.
*Paeonia lactiflora* (Peony) *Paeonia lactiflora* is used as an adjunctive treatment for respiratory disorders due to its antispasmodic, anti-inflammatory, and expectorant properties, helping reduce cough and airway irritation [78].	Paeoniflorin: The major biologically active monoterpene glycoside isolated from peony roots. In traditional medicine, it is valued for its anti-inflammatory, analgesic, neuroprotective, and immunomodulatory activities. Paeonol: a phenolic compound with antioxidant, anti-inflammatory, and mild antimicrobial properties. Paeoniflorin inhibits NF-κB, modulates MAPK, and activates Nrf2/ARE signaling. Paeonol blocks NF-κB and MAPK inflammatory pathways.
*Pelargonium sidoides* (African Geranium) *Pelargonium sidoides* roots are used for colds and bronchitis. The plant exhibits antibacterial, antiviral, immunomodulatory, and mucolytic effects and may reduce respiratory inflammation and symptoms [79,80,81].	Paeonol: a phenolic compound with antioxidant, anti-inflammatory, and mild antimicrobial properties. Coumarins (e.g., umckalin): prevent bacterial adhesion to the respiratory mucosa. Flavonoids (quercetin and kaempferol): exhibit pronounced antioxidant and anti-inflammatory activities. Tannins: produce astringent effects, reduce tissue swelling, and protect mucosal surfaces. Phenolic acids: provide antibacterial and immunomodulatory effects. Quercetin and kaempferol inhibit NF-κB-mediated inflammatory signaling and reduce the production of pro-inflammatory cytokines such as TNF-α, IL-1β, and IL-6. They also suppress COX-2 and LOX enzyme activity and modulate MAPK pathways. In addition, they enhance endothelial nitric oxide (NO) bioavailability, contributing to vasodilation and vascular protection, and activate antioxidant defense mechanisms, including the Nrf2 pathway.
*Uncaria tomentosa* (Cat’s Claw) *Uncaria tomentosa* contains oxindole alkaloids and polyphenols with antiviral, anti-inflammatory, and immunomodulatory effects. It may reduce symptoms of respiratory viral infections and bronchitis and relieve bronchial spasms [82].	Oxindole alkaloids (e.g., isopteropodine and mitraphylline): stimulate phagocytosis and regulate T-lymphocyte activity. Polyphenols and flavonoids: exert strong antioxidant effects and reduce oxidative stress in lung tissues. Tannins and proanthocyanidins: possess marked antimicrobial and antifungal activities. Triterpenes and phytosterols: suppress inflammatory processes at the cellular level, reducing edema and pain in a manner similar to nonsteroidal anti-inflammatory drugs. Inhibits NF-κB, reducing TNF-α, IL-1β, IL-6. Immunomodulatory (macrophages, T-cells, phagocytosis). Antioxidant via ROS scavenging and Nrf2 activation. Inhibits COX/LOX pathways (↓ prostaglandins, leukotrienes).
*Thymus vulgaris* (Thyme) Thyme preparations are used as expectorants for cough and upper respiratory disorders. Thymol may reduce bronchial smooth muscle spasms by modulating calcium and potassium channels [83].	Thyme contains 0.1–0.6% essential oil, with thymol as the major constituent (up to 30%), together with carvacrol. It also contains tannins, bitter compounds, minerals, gums, organic pigments, and the triterpenoids ursolic and oleanolic acids. Antioxidant: thymol and carvacrol scavenge ROS and reduce oxidative stress. Anti-inflammatory: inhibition of NF-κB signaling and downregulation of TNF-α, IL-1β, IL-6. COX/LOX inhibition: reduces prostaglandin and leukotriene synthesis Antimicrobial: disruption of bacterial and fungal cell membranes by essential oils. Spasmolytic/expectorant: relaxation of bronchial smooth muscle and enhanced mucus clearance.
*Hedera helix* (Common Ivy) Ivy leaf extracts are widely used as herbal cough remedies due to their expectorant properties and have been applied in the management of chronic airway obstruction and bronchial asthma [79].	Hederin is the principal active constituent of ivy preparations. It enhances bronchial gland secretion, promotes mucus liquefaction, and facilitates sputum expectoration in both productive and nonproductive cough. Expectorant: increases bronchial secretions and mucus clearance (mainly via hederacoside C to hederin). Bronchospasmolytic: relaxes airway smooth muscle (β2-adrenergic activity). Anti-inflammatory: reduces NF-κB-mediated cytokine production Mucolytic support: decreases mucus viscosity and improves cough productivity.
*Plantago* spp. (Plantain) *Plantago* spp. exhibit anti-inflammatory, antimicrobial, and expectorant properties. Mucilage protects the respiratory mucosa, while aucubin and flavonoids may reduce inflammation and bronchial irritation [84].	Aucubin is a naturally occurring iridoid glycoside found primarily in plantain leaves and several medicinal plants. It is valued for its antibacterial, anti-inflammatory, antioxidant, and hepatoprotective properties. Anti-inflammatory: suppresses NF-κB signaling and pro-inflammatory cytokines (TNF-α, IL-1β, IL-6). Mucilaginous protective effect: polysaccharides form a protective film on mucosa (demulcent action). Antimicrobial: aucubin and phenolic compounds inhibit bacterial growth. Antioxidant: flavonoids and iridoids scavenge ROS and reduce oxidative stress. Wound healing: promotes epithelial regeneration and tissue repair.
*Primula veris* (Cowslip) *Primula veris* is traditionally used as an expectorant for cough and respiratory disorders. Root preparations help liquefy bronchial secretions and soothe inflamed airways [85], p. 221.	Primula leaves contain up to 5.9% ascorbic acid and saponins and are commonly used as herbal teas for vitamin deficiency. The rhizomes and roots contain up to 10% triterpene saponins, whose aglycones include primulagenins A, D, and SD. Expectorant: triterpene saponins stimulate bronchial secretion and mucus clearance. Anti-inflammatory: inhibition of NF-κB-mediated cytokines (TNF-α, IL-1β, IL-6). Mucolytic/secretolytic: reduces sputum viscosity and improves airway drainage. Antimicrobial: saponins and flavonoids show mild antibacterial activity. Mild sedative/analgesic: flavonoids contribute to calming and antispasmodic effects.
*Sambucus nigra* (Black Elderberry) Elder flowers and berries are traditionally used for colds, influenza, and upper respiratory infections. Their anti-inflammatory and antiviral effects are associated with antioxidant compounds, particularly anthocyanidins [86,87].	Anthocyanins: potent plant pigments responsible for the dark coloration of fruits and their strong antioxidant activity. Flavonoids (including rutin and quercetin): reduce inflammation, strengthen vascular walls, and scavenge free radicals. Antiviral: inhibits viral entry and replication (polyphenols, anthocyanins). Anti-inflammatory: suppresses NF-κB signaling and reduces TNF-α, IL-1β, IL-6. Antioxidant: strong ROS scavenging due to anthocyanins and flavonoids. Immunomodulatory: enhances cytokine response and immune cell activation. Diaphoretic/expectorant: supports mucus clearance and sweating response in respiratory infections.
*Arctium lappa* (Burdock) Burdock root exhibits anti-inflammatory activity, mainly attributed to lignans such as arctigenin, which inhibit nitric oxide production in macrophages [88].	Lignans are among the most pharmacologically important constituents of burdock. These include podophyllotoxin, arctiin, matairesinol, 7-hydroxymatairesinol, secoisolariciresinol diglucoside, and secoisolariciresinol. Some lignans exhibit antitumor, hepatoprotective, estrogen-like, antioxidant, and central nervous system-stimulating activities [89]. Anti-inflammatory: inhibits NF-κB signaling and reduces TNF-α, IL-1β, IL-6. Antioxidant: lignans and polyphenols scavenge ROS and activate Nrf2 pathways. Antimicrobial: phenolic compounds and lignans inhibit bacterial growth. Detoxifying/hepatoprotective: supports liver antioxidant enzymes and xenobiotic metabolism. Immunomodulatory: modulates macrophage activity and cytokine balance.
*Origanum vulgare* (Oregano) Oregano is used in herbal formulations for respiratory infections due to its anti-inflammatory and expectorant properties. It may also stimulate lymphatic flow and drainage [85] (p. 186), [90,91].	*Origanum vulgare* contains tannins, ascorbic acid, thymol, carvacrol, bi- and tricyclic sesquiterpenes, and geranyl acetate. Oregano extracts and essential oils suppress the production of pro-inflammatory mediators. Anti-inflammatory: inhibits NF-κB signaling and reduces TNF-α, IL-1β, IL-6. Antioxidant: thymol, carvacrol, and phenolics scavenge ROS. Antimicrobial: strong antibacterial and antifungal activity via membrane disruption. COX/LOX inhibition: reduces prostaglandin and leukotriene synthesis. Spasmolytic: relaxes smooth muscle (digestive and respiratory tract).
*Verbena officinalis* (Vervain) *Verbena officinalis* is traditionally used for respiratory infections and exhibits expectorant, anti-inflammatory, antiviral, and antispasmodic properties, helping relieve cough and bronchial inflammation [92,93].	*Verbena officinalis* contains bitter compounds, the lactone glycoside verbenalin, tannins, alkaloids, and carotene. Anti-inflammatory: inhibits NF-κB signaling and reduces TNF-α, IL-1β, IL-6. IL-17A modulation: verbenalin suppresses IL-17A-mediated inflammation. Antioxidant: flavonoids and iridoids scavenge ROS COX/LOX inhibition: decreases prostaglandin and leukotriene synthesis. Immunomodulatory: regulates macrophage and lymphocyte activity.
*Crataegus almaatensis* (Hawthorn) *Crataegus almaatensis* contains flavonoids with cardioprotective, vascular, and anti-inflammatory effects. Experimental studies also demonstrated an approximately 50% increase in lymph flow [94].	Hawthorn exerts its biological effects through a rich complex of bioflavonoids (quercetin, hyperoside, and vitexin), triterpenoids (ursolic and oleanolic acids), and phenolic acids. These compounds modulate the activity of enzymes, ion channels, and cellular receptors. Antioxidant (flavonoids, phenolic acids). Cardioprotective (improves myocardial metabolism, vascular function). Anti-inflammatory via NF-κB inhibition. Vasodilatory (endothelial NO modulation).
*Ziziphora bungeana* The antioxidant and vasoprotective properties of *Ziziphora bungeana* are attributed to its flavonoid profile, including apigenin, chrysin, acacetin, and linarin. Additional bioactive compounds such as betulin, β-sitosterol, and the rare triterpene 3β-acetoxyolean-11-en-28,13β-olide have been associated with modulation of inflammatory processes, vascular reactivity, and lymphatic drainage. In our experiments, Z. bungeana increased lymph flow by 43% [93,95].	The biological activity of *Ziziphora bungeana* is attributed to its rich phytochemical composition, characterized by high concentrations of terpenoids (up to 73% pulegone) and flavonoids, particularly luteolin and apigenin. Antioxidant and anti-inflammatory (luteolin, apigenin, terpenoids). NF-κB inhibition and cytokine reduction. Antimicrobial (essential oil rich in pulegone). Smooth muscle relaxant (spasmolytic).
*Echinacea purpurea* *Echinacea purpurea* possesses antimicrobial and anti-inflammatory properties and may enhance lymphatic drainage, supporting its potential role in regulating lymphatic function [96].	Polysaccharides (arabinogalactans, heteroglycans): immunomodulatory, enhance macrophage activity and phagocytosis. Alkamides (e.g., echinacein): anti-inflammatory, interact with CB2 receptors. Caffeic acid derivatives (especially chicoric acid, also chlorogenic and caffeic acids): strong antioxidant and immune-supporting effects. Flavonoids (quercetin, kaempferol, isorhamnetin): antioxidant and capillary-protective activity. Glycoproteins: stimulation of innate immune responses. Polyacetylenes: mild antimicrobial activity Essential oil components (minor sesquiterpenes/monoterpenes): weak antimicrobial effects. Immunomodulatory (macrophage activation, phagocytosis). Modulates cytokines (TNF-α, IL-1β, IL-6). Anti-inflammatory via NF-κB inhibition. Antioxidant (caffeic acid derivatives, flavonoids).
*Bergenia crassifolia* Preparations of *Bergenia crassifolia* possess hemostatic, astringent, anti-inflammatory, hepatoprotective, and antimicrobial properties. They strengthen vascular walls, moderately lower blood pressure, slightly increase heart rate, and enhance lymph flow by approximately 40% [93].	Tannins are present in high concentrations in *Bergenia crassifolia* (15–27% in rhizomes and 13–23% in leaves) and are responsible for its astringent, anti-inflammatory, and hemostatic properties. Bergenin (an isocoumarin glycoside): a characteristic constituent of bergenia with antimicrobial, antioxidant, diuretic, and wound-healing activities. Arbutin: a phenolic glycoside with pronounced antiseptic and anti-inflammatory properties. Flavonoids (quercetin and hyperoside): plant polyphenols that strengthen blood vessels and protect cells against oxidative damage. Strong antioxidant (bergenin, tannins). Anti-inflammatory (NF-κB inhibition). Antimicrobial and astringent (high tannin content). Hemostatic and tissue-protective effects.
*Hedȳsarum neglēctum* The roots of *Hedysarum neglectum* contain flavonoids, catechins, saponins, tannins, xanthines, and alkaloids. The plant exhibits anti-inflammatory, antitumor, immunostimulatory, and tonic activities, while some species of the genus have demonstrated antiviral and antibacterial properties. Our previous studies showed that *H. neglectum* stimulates interstitial humoral transport and increases lymph flow by approximately 50% [97,98].	Flavonoids such as mangiferin, quercetin, and hyperoside exhibit strong antioxidant activity, strengthen vascular walls, and alleviate spasms. Catechins (tannins): possess potent anti-inflammatory activity and inhibit bacterial growth. They are also responsible for the characteristic red coloration of root infusions. Triterpene saponins: promote expectoration and facilitate mucus clearance. Coumarins: contribute to thrombosis prevention and suppression of inflammatory processes. Antioxidant: flavonoids and phenolic compounds scavenge ROS. Anti-inflammatory: inhibits NF-κB-mediated cytokine production. Immunomodulatory: supports macrophage and lymphocyte activity. Vascular-protective: strengthens capillary walls (polyphenols).
*Chamaenerion angustifolium* (Fireweed, Ivan Tea) *Chamaenerion angustifolium* is traditionally used for respiratory disorders. It may reduce inflammation and cough, while facilitating sputum liquefaction and expectoration in bronchitis and tracheitis.	The leaf biomass of *Chamaenerion angustifolium* contains organic acids, tannins, anthocyanins, carotenoids, flavonoids, ascorbic acid (25.15–49.11 mg%), pectin, rutin, polysaccharides, lignin, coumarins, aurones, sterols, triterpenes, simple phenols, polyphenolic compounds, and glycosides. The antioxidant activity of the plant has been strongly associated with its high polyphenol content [99]. Anti-inflammatory: suppresses NF-κB signaling and cytokines (TNF-α, IL-6, IL-1β). Antioxidant: high polyphenol and flavonoid content (strong ROS scavenging). Antimicrobial: tannins and phenolics inhibit bacterial growth. Astringent & tissue-protective: reduces inflammation and promotes mucosal repair.

↑ indicates an increase or upregulation; ↓ indicates a decrease or downregulation.

## Data Availability

Data are not available for sensibility/patent/copyright/privacy/legal/data protection/ethical reasons.
